# Mindfulness as an intervention to lower blood pressure

**DOI:** 10.1038/s41371-026-01187-w

**Published:** 2026-08-06

**Authors:** James E. Sharman

**Affiliations:** Menzies Institute for Medical Research, University of Tasmania, GPO Box 341, Hobart, TAS 7001 Australia

**Keywords:** Health care, Risk factors

The usual pathway towards development of hypertension include a range of behavioural traits that are potentially within the control of individuals to modify and lower the future risk of developing hypertension and its harmful cardiovascular disease consequences. Most hypertension can be prevented or delayed, or if already present, can be improved, by consistent adoption of healthy behaviours such as maintenance of a heart-healthy eating pattern with low salt intake, normal body weight without obesity, regular physical activity and moderation of alcohol. The benefits of maintaining these lifestyle elements are backed by strong evidence and recommended in hypertension guidelines globally [1–3].

Another potential lifestyle element to tackle hypertension is lowering psychological stress. Guidelines are generally consistent, but cautious, to acknowledge that relaxation techniques have a likely role to lower blood pressure [1–3]. A wide variety of techniques can be employed, including mindfulness meditation, controlled breathing exercises, movement-based practices such as yoga, biofeedback to train autonomic control or psychological stress reduction methods, to name just a few categories of possible interventions. The appeal of relaxation techniques is their widespread availability, mostly at low cost, and that they can be used as adjuncts to pharmacotherapy, with the perception that there is low risk of harm. Currently, relaxation techniques are not recommended as primary therapy for hypertension, due a lack of high quality evidence [4].

To advance the field, there is a need to consolidate evidence to understand with more certainty about the blood pressure-lowering effects of specific relaxation techniques, especially those that can be pragmatically implemented. Efforts to consolidate evidence on mindfulness-based interventions have recently been undertaken by Dr Lee and colleagues [5], where in this issue of the *Journal*, they present findings from an umbrella review of systematic reviews and meta-analyses of randomised controlled trials on blood pressure in adults with or without hypertension. This type of review is considered as the highest level of evidence and most useful when many systematic reviews exist on a particular intervention. The authors examined 11 systematic reviews and meta-analyses on blood pressure effects from mindfulness-based interventions, and this also included assessment of the methodological quality and overall strength of evidence for each of the systematic reviews [5].

The primary findings were a consistent blood pressure lowering effect of mindfulness-based interventions, approximating −6 mmHg for systolic and −3 to −5 mmHg for diastolic blood pressure. These are clinically meaningful effects of similar magnitude to other lifestyle interventions. However, there were wide confidence intervals for the effect estimates and notable methodological caveats, including most of the reviews were deemed to be of critically low quality, with small sample sizes, heterogenous participant characteristics, short term follow ups (e.g. ≤12 weeks) and under reporting of safety outcomes. Moreover, and somewhat ironically, even though the umbrella review was focussed on a single type of relaxation technique – mindfulness-based interventions – there remained sizable diversity among the type of interventions, as well as the control comparators. The overall certainty of evidence was rated as low or very low.

From these data, the authors recommend that more high-quality, larger trials of mindfulness-based interventions are needed before it can be recommended as routine treatment for hypertension. While this is consistent with the prevailing messaging already within some hypertension guidelines, the work emphasises critical gaps, including the need for better assessment of safety outcomes, more consistency in the assessment of blood pressure, and the need for longer term follow up to determine sustainability of interventions beyond 12 months, as well as effects on cardiovascular disease outcomes. Crucially, the wide diversity in the type of mindfulness-based interventions is a problem that amplifies variability in effect estimates and will continue to create uncertainty unless addressed. Although this diversity is reflective of mindfulness-based interventions being heterogenous by design, expert consensus may be helpful to agree upon a standardised protocol for people with hypertension.

A recent trial led by Professor Loucks, who is part of the umbrella review’s author team, tested a mindfulness-based stress-reduction program adapted for people with hypertension. The results show promising effects on lowering blood pressure [6]. This program involves standardised education about risk factors for hypertension and mindful awareness of diet, physical activity, medication adherence, alcohol consumption, stress, and social support for behaviour changes to lower blood pressure [6]. While results are encouraging, the program is multifaceted, requiring expertise from trained instructors to deliver mindfulness training and hypertension education, as well as substantial time commitment by participants. The magnitude of resourcing needed for effective delivery of a mindfulness-based program are not dissimilar from other lifestyle interventions (i.e. supervised exercise training), but nonetheless it will be important to understand cost effectiveness of implementation. Finally, the mindfulness-based stress reduction program may also need further adapting for broader external validity, for example to achieve greater reach to disadvantaged populations, as it has primarily been tested among well-educated, White participants [6].

For individuals, making sustainable changes to any lifestyle habits for better health requires ongoing commitment and persistent assimilation of new habits into daily life. For people with hypertension wanting to know what they can do to lower their blood pressure beyond taking antihypertensive medications, on balance the evidence continues to provide cautionary support of practicing mindfulness to reduce psychological stress. Whilst not yet ready for routine clinical implementation, the important work from Dr Lee et al. [5] helps to focus future intent to address evidence gaps and more fully understand how mindfulness-based interventions may best applied to achieve the greatest benefit for people with hypertension.
